# Classical biological control of the brown marmorated stink bug (*Halyomorpha halys*) in apple orchard: a success story

**DOI:** 10.1002/ps.70154

**Published:** 2025-08-25

**Authors:** Claudio Ioriatti, Valerio Mazzoni, Pietro Franceschi, Serena G Chiesa, Gino Angeli, Matteo De Concini, Claudio Panizza, Fabrizio Martini, Livia Zapponi, Gianfranco Anfora

**Affiliations:** ^1^ Fondazione Edmund Mach, Research and Innovation Centre Trento Italy; ^2^ Fondazione Edmund Mach, Technology Transfer Centre Trento Italy; ^3^ Associazione Produttori Ortofrutticoli Trentini Trento Italy; ^4^ National Research Council of Italy, Institute of BioEconomy Trento Italy; ^5^ Center for Agriculture, Food and Environment (C3A), University of Trento Trento Italy

**Keywords:** Brown marmorated stink bug, parasitoid, *Trissolcus japonicus*, invasive species, pest, fruit damage

## Abstract

**BACKGROUND:**

The brown marmorated stink bug (BMSB), *Halyomorpha halys*, is an invasive pest from eastern Asia that has caused significant damage to apple orchards in Europe and the United States. To reduce pesticide reliance, a classical biological control (CBC) program, using the Asian egg parasitoid *Trissolcus japonicus*, was initiated in Trentino, a key apple‐producing area in Italy. The CBC program involved the release of *T. japonicus* at selected sites with minimal chemical input from 2020 to 2023. Monitoring was conducted to assess parasitoid establishment and egg parasitization rates. Preliminary data on fruit damage were also collected to assess trends at a large territorial scale. Parasitoid efficacy was evaluated based on discovery efficiency and parasitization impact.

**RESULTS:**

*Trissolcus japonicus* was recaptured in 62.3% of the sites, with increasing recapture rates over the years (from 30% in the first year to 65.4% in the fourth year). The overall egg parasitization rate increased from 18.3% to 50.6%, with *T. japonicus* contributing significantly (from 7.9% in 2020 to 41.3% in 2023). The percentage of hatched eggs decreased from 61.6% to 29.0%. Discovery efficiency improved from 9.9% to 54.4%, and impact from 7.9% to 39.0%. Fruit damage monitoring indicated an overall decreasing trend in orchards within a 2.5 km radius of successful release sites.

**CONCLUSION:**

The release of *T. japonicus* in Trentino demonstrated effective control of BMSB, reducing pesticide use and fruit damage, thereby highlighting the potential of CBC as a sustainable pest management strategy against invasive alien pests. Further long‐term studies are recommended to optimize and expand this approach. © 2025 The Author(s). *Pest Management Science* published by John Wiley & Sons Ltd on behalf of Society of Chemical Industry.

## Graphical abstract

The classical biological control program with *Trissolcus japonicus* in Trentino effectively reduced *Halyomorpha halys* damage in apple orchards, increasing discovery efficiency and egg parasitization rates from 2020 to 2023. The results demonstrate reduced pesticide use and fruit damage, highlighting CBC as a sustainable pest management strategy against invasive pests.

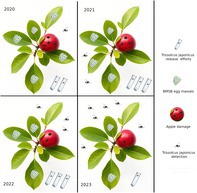

## 1 INTRODUCTION

Trentino is an autonomous province located in the northeast of Italy (centroid: 46°6′13″ N, 11°4′22″ E) and with its 10 000 ha of apple orchard, is one of the most important districts for apple production. The brown marmorated stink bug (BMSB), *Halyomorpha halys* (Stål, 1855) (Hemiptera: Pentatomidae) was first detected in Trentino in 2016. Since then, the pest has gradually spread first within the valley floor orchards and then into the low, middle, and upper hills of the apple growing area, which were reached in 2019.^1^, ^2^

BMSB is a polyphagous, invasive insect of economic concern to agricultural production. Native to eastern Asia, BMSB was first recorded outside its native range in the United States in the mid‐1990s and in Europe almost 10 years later. Since then, it has become one of the most serious pests of (Italian) fruit orchards, including apple.^3^ Despite the number of recently developed IPM tools and tactics, such as multi‐modal trapping,^4^ trap crops,^5^ push and pull^6^, ^7^ exclusion nets,^8^, ^9^ long‐lasting insecticide‐treated nets,^10^, ^11^ sterile insect technique^12^ and behavioral manipulation,^4^ BMSB control in apple orchards still relies heavily on pesticide application.^13^, ^14^

Considerable investment has been also made in the development of effective monitoring tools,^15^, ^16^ but despite the significant improvement in their reliability,^4^, ^17^, ^18^ management of this pest still requires a high frequency of treatments, resulting in overuse of chemicals that has contributed to the discontinuation of low‐impact IPM practices or secondary outbreaks.^19^

In addition, the habitat composition of agricultural landscapes is a key factor determining the dynamics of this pest in agroecosystems. Semi‐natural habitats are important elements in sustaining BMSB populations and consequently determining orchard colonization.^20^ In fact, large populations of BMSB overwinter outside the orchard and grow on wild host plants common in woodlands that often border orchards,^21^ from which they invade the orchard following a seasonal dynamic.^22^, ^23^, ^24^

This behavior has prompted researchers to reflect on the importance of controlling populations in non‐agricultural habitats with an area wide approach through biological control services in the landscape and consequently relieving infestation pressure on the crop.^25^

BMSB is an alien species that is not effectively controlled by the native beneficial faunal assemblage of newly invaded areas.^26^, ^27^ On the other hand, the recent increase in alien species invasions has revitalized interest in classical biological control (CBC) as a sustainable management strategy in general and for the specific case of BMSB as well. CBC can be an effective strategy to control invasive insect pests^28^ and can offer protection to the natural ecosystem.^29^ To this end, *Trissolcus japonicus* (Ashmead) (Hymenoptera: Scelionidae), an Asian egg parasitoid of the BMSB, was identified as the most promising biocontrol agent (BCA). In order to propose *T. japonicus* as a candidate classical biological control agent against BMSB, its biosafety has been evaluated in quarantine facilities in the United States,^30^, ^31^ Europe^27^, ^32^ and New Zealand^33^ aiming to assess its efficacy on BMSB and its host‐range with regard to the pentatomid indigenous fauna. Incidentally, resident egg parasitoid surveys conducted in the United States and Europe have often recorded adventive populations of *T. japonicus*.^34^, ^35^, ^36^ In Trentino, adventive populations of *T. japonicus* were detected only in one location the year before the release program began.^21^, ^22^, ^23^, ^24^, ^25^, ^26^, ^27^, ^28^, ^29^, ^30^, ^31^, ^32^, ^33^, ^34^, ^35^, ^36^, ^37^ The same studies indicated that adventive populations of another alien parasitoid, *Trissolcus mitsukurii* (Ashmead), were also present in the region and that among native species, *Anastatus bifasciatus* (Geoffroy) was identified as the predominant parasitoid of BMSB. In 2020, after the evaluation of a comprehensive risk assessment submitted by Italian scientific institutions and phytosanitary services, the Italian Ministry of Ecological Transition authorized the release of *T. japonicus* in five regions and two autonomous provinces within the framework of a national biological control program.^19^

The short‐term establishment and efficacy of *T. japonicus* in Trentino‐South Tyrol region were previously investigated, with results demonstrating that the parasitization rate increased significantly over time compared to the control sites.^19^ However, these rates were still lower than those recorded in its native range in northern China,^38^ suggesting potential for further increase in the future.

In the present study, conducted using a wide‐area approach, we aim to assess the efficacy of the biological control program over a longer period (4 years), testing the hypothesis of a potential further increase in parasitization rates. Additionally, we seek to evaluate the impact on fruit damage at harvest and the implementation of pesticide programs at the apple production district level, two aspects not addressed in the previous study.

## 2 MATERIALS AND METHODS

### 2.1 Apple production system

The apple growing area of the Trentino province could be divided into two distinct geographical sub‐areas: the hilly part accounting for about 6500 ha and the valley floor where the 3500 ha of apple orchards are mixed with vineyards and other fruit crops. The production system is characterized by small and fragmented farms, whose average size is 1.2 ha. To overcome these structural challenges, Trentino apple growers have turned to cooperatives, which associate 85% of producers and market 90% of apple production.

In this context, guidelines for Integrated Apple Production have been implemented since 1991^37^ within the conceptual framework of the IOBC guidelines.^39^ A technical advisory service provided by Fondazione Edmund Mach (FEM) supports growers and cooperatives distributing plant protection products in making decisions with an area‐wide approach^40^ on what/when/where to apply pesticides based on field scouting, fruit sampling, pest thresholds and pest forecasting models.

### 2.2 BMSB chemical control

Since 2019, a steering committee involving delegates from advisory services (FEM and private), FEM researchers and cooperative representatives has been formed to decide on the BMSB control strategy based on the reported data on weekly catches in BMSB traps (Rescue trap‐Sterling International, Inc., Spokane, WA, USA), baited with the aggregation pheromone (Trécé Inc. Dual Lure) sampling insects with the beating method and estimating fruit infestation with visual fruit inspections in orchards.

Afterward, growers were advised on the correct timing of insecticide application or, in cases where the infestation was not widespread, were asked to check the actual population of BMSB in their own orchards by scouting for injured fruits and to act accordingly.

The management of BMSB in IPM orchards predominantly relied on specific treatments with acetamiprid (*e.g*., Epik SL, Kestrel, and Gazelle) as the first choice. However, other insecticides registered for BMSB control were included in the seasonal control strategy when needed (Table 1S). Pest control also benefited from the collateral effect of other synthetic insecticides potentially used throughout the season to control other pests (*e.g*. codling moth (*Cydia pomonella* L.) and Mediterranean fruit fly (*Ceratitis capitata* Wiedemann)).

The number of specific treatments per hectare and the percentage of apple orchard surface treated for controlling BMSB during the 4‐year program were calculated based on annotations from digital farm field books belonging to apple grower members of the cooperative organization. The number of field books (farms) analyzed ranged from 3940 in 2019 to 3715 in 2023 and corresponded to a surface fluctuating between 8196.2 and 8122.3 ha (Table S2). These data are centrally stored by the cooperative organization and was analyzed for the purposes of the present study. The decrease in the number of farms recorded during the period considered is attributable to the ongoing trend of expanding farm areas following the abandonment by some fruit growers.

### 2.3 CBC program: site selection and release procedure

From 2020 to 2023, the province of Trento participated in a national CBC program involving the release of *T. japonicus* to manage BMSB populations. The *T. japonicus* release sites were selected after field inspections aimed at identifying the presence of BMSB individuals and/or attractive host plant species. To improve the probability of establishment, only sites with minimal or no chemical input were chosen. The parasitoids used for the releases were provided by CREA‐DC (Consiglio per la ricerca in agricoltura e l'analisi dell'economia agraria – Difesa e Certificazione, Italy) and reared in FEM on BMSB egg masses from specifically maintained colonies. Following a national protocol for *T. japonicus* release, the individuals were placed in plastic tubes (VWR 50‐mL centrifuge tubes, 525‐0611) with a drop of honey, and maintained in climatic chambers at 24 °C ± 1 °C, 60 ± 5% relative humidity (RH), and a 16 h: 8 h photoperiod. At each site, three releases of 100 females and 10 males, 1–3 weeks old, were performed 3 weeks apart, from the end of June to mid‐August each year.^18^

During the 4 years of the release program, 53 sites were involved, with parasitoid releases conducted for one (21 sites), two (23 sites), or three (nine sites) years. Four sites (three in the second year and one in the third year) were dropped after the first release because they were considered unsuitable for establishment as no BMSB egg masses were found. In 21 sites, parasitoid release was stopped after detection, but monitoring continued to evaluate further development of the population (Fig. S1).

In none of the selected release sites, the presence of *T. japonicus* was detected before its initial release. A minimum distance of 1 km was maintained between neighboring release sites and the single site where adventive populations of *T. japonicus* had been documented.^36^

### 2.4 Sampling protocol and morphological identification

The surveys were conducted once before and twice after the release of the biological control agent *T. japonicus*. In all years, the pre‐release sampling took place in June, while the two post‐release samplings were conducted from early August to mid September, when the highest population density of BMSB was expected. The exact sampling dates varied slightly each year to adapt to weather conditions and seasonal earliness. The sampling protocol involved a visual inspection of all vegetation (herbs, shrubs, and trees), from the ground to a height of 2 m, within a 50‐m radius for 1 h per session, targeting egg masses of both Pentatomidae and Acanthosomatidae, a closely related family. The leaves with egg masses were collected, stored in plastic tubes, and brought to the laboratory for further analysis. Morphological identification of collected egg masses was made with a stereomicroscope and determined to species or genus level according to reference taxonomic keys.^41^, ^42^, ^43^ Single eggs were categorized into four groups: hatched, unhatched, predated, and parasitized. For BMSB, emergence holes were identified according to their shape and morphology of meconium^44^ to species or family level. Emerged parasitoids were transferred to 70% ethanol and morphologically identified using a stereomicroscope and the taxonomic keys.^19^

### 2.5 Parasitoid efficacy

Two indices of parasitoid efficacy were calculated for the collected egg masses: ‘Discovery efficiency’ and ‘Parasitoid impact’.^45^ The ‘discovery efficiency’ describes the parasitoid's ability to find the egg masses, calculated as the number of egg masses with at least one parasitized egg divided by the total number of collected egg masses. The parasitoid impact was calculated as the number of parasitized eggs by *T. japonicus* (through the identification of emerged individuals and according to meconium morphology^46^ divided by the total number of collected eggs. These analyses were performed for BMSB egg masses collected in all the monitored sites. For the ‘Parasitoid impact’ analysis, only egg masses yielding a single parasitoid species were included. This was because DNA‐based approaches^47^ were necessary to determine parasitism of unhatched eggs, and excluding egg masses with multiple species prevented under−/overestimation of parasitism rates due to intrinsic competition or hyperparasitism.

### 2.6 Fruit damage

The fruit damage dataset was not directly designed to test the efficacy of the CBC control strategy, but was extracted from the standard field monitoring activities performed at the territorial scale by the extension service of the Fondazione E. Mach. The monitoring protocol relies on the sampling of a variable number of apples (from 100 to 1000) in each orchard recording the number of fruits showing visible BMSB damage. From this dataset, which includes observations collected from 2019 to 2023 (# 2345 observations), we selected a subsample of data relative to fruit orchards located within a geographic buffer of 2.5 km radius around the release points where the parasitoid had been established. Superimposed buffers were merged into areas and to maximize statistical robustness we restricted the analysis to five areas showing more than 100 observations (Table 1). The five areas were located at different altitudes, ranging from 83.5 to 581 m above sea level, and hosted different population sizes that in part depended on when the BMSB infestation began. In addition, within each area the analysis was performed on the most represented apple varieties, also discarding those which showed sparse sampling. A table showing the number of samples included in the statistical analysis is included in the Supporting Information (Table S3).

**Table 1:** Relevant characteristics of the areas where *T. japonicus* has been established and where fruit sampling for injury levels was performed

Area	Apple district	Surface (Km^2^)	Number of samples	Average altitude (m asl)	Infestation description
#1	Arco	20	72	83.5	Located in the Sarca valley. Presence of mixed crops. Highly infested since the first years of the invasion
#2	Low Non Valley	17.4	161	431.0	Located in the lower part of Non Valley. Intensive apple growing area. Infestation was still spreading during the period considered
#3	Middle Non Valley	31.5	335	581.0	Located in the middle part of Non Valley. Intensive apple growing area with the lowest level of infestation during the period considered
#4	Trento North	36	166	201.0	Located in the Adige valley floor. Presence of mixed crops, mainly grape and apple. Highly infested since the first years of the invasion
#5	Trento South	28	195	183.0	Located in the Adige valley floor. Presence of mixed crops, mainly grape and apple. Highly infested since the first years of the invasion

The different apple varieties had different ripening times: mid to late August (‘Gala’), late August to mid‐September (‘Golden Delicious’ and ‘Red Delicious’), mid to late September (‘Granny Smith’), and late September (‘Fuji’). The surveys were performed in 2 months preceding the harvest of each cultivar.

### 2.7 Statistical analysis

To analyze the trend of parasitism rates in observed stink bug egg masses from 2020 to 2023, we employed the Cochran‐Armitage proportion test for trend. The data were analyzed using R statistical software,^48^ employing the DescTools package. This test was chosen for its capability to detect linear trends in proportions across ordinal categories, such as the years in our study.^49^ When making multiple comparisons involving different parasitoids on the total number of observed eggs, the Bonferroni principle was applied.

All statistical analyses concerning fruit injury were performed in R relying on the tidyverse, sf and terra for spatial data manipulation and visualization.^50^, ^51^, ^52^ Statistical modeling of the data collected on the five individual areas was performed relying on the mgcv package,^53^ while the gratia package was used for model visualization and checking.^54^ The adjusted predictions from the models which were used to highlight the effect of time and variety on the response variable were calculated with the marginaleffects package.^55^

### 2.8 Modeling strategy

The number of damaged apples inside each orchard was modeled as a function of the following predictors:

● number of sampled fruits: accounts for the different size of sampling

● latitude/longitude: to take into account the anisotropy in the distribution of the damage and of the sampling

● apple variety

● year: represented as a categorical variable

● day of the year (doy): this predictor was mean centered to make the intercept of the model more interpretable

● gst: growing season average temperature calculated from a 16‐year average of MODIS surface temperature satellite data

A Generalized Additive Negative Binomial Model (GAM) with the following structure was fit on the individual areas:


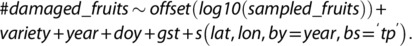


GAMs^55^ combine the flexibility of non‐parametric models with the interpretability and robustness of generalized linear models. The structure of the model was manually tuned, striking a balance between model complexity and level of fit taking into consideration the AIC of the individual models. The smooth term, which accounted for the spatial anisotropy in the sampling was allowed to vary across the years (*by = year*) to take into consideration the variability of the sampling sites. In all cases but one, the model accounted for around 50% of the deviance. The overall quality of the fit was always checked with the standard diagnostic plots which are included in the Supporting Information (Fig. 2S).

## 3 RESULTS

### 3.1 Parasitoid establishment

During the four‐year program, *T. japonicus* was released at a total of 53 sites.

- In Year 1, 20 sites were selected for the release, all of which were monitored. *T. japonicus* was detected at six sites (30.0% of the monitored sites).
- In Year 2, *T. japonicus* was released at 42 sites, including 27 new release sites and 15 from Year 1. Two Year 1 sites were excluded as they were deemed unsuitable for establishment, while three other sites where the parasitoid had been detected in Year 1 were not included in the new release program but continued to be monitored. *T. japonicus* was monitored in 44 sites and it was detected at 14 sites (31.8%).
- In Year 3, *T. japonicus* was released at 43 sites, six of which were new. Of the previous year's sites, one was excluded, and four sites that had hosted the parasitoid in previous years did not receive any additional releases. A total of 48 sites were monitored, and *T. japonicus* was detected at 26 sites (54.2%).
- In Year 4, due to funding restrictions, *T. japonicus* was released at only 10 sites, with no new release sites selected. The parasitoid was monitored at 26 sites, 21 of which had hosted *T. japonicus* in previous years, while five had never detected the parasitoid before. *T. japonicus* was detected at 17 of the 26 monitored sites (65.4%) (Table 2).

**Table 2:** Number of sites included in the release and monitoring program over the 4‐year period and number and percentage of sites where *T. japonicus* was recaptured

Year	Release sites	Monitored sites	Sites where *T. japonicus* was recaptured	
	n.	n.	n.	%
2020	20	20	6	30,0
2021	42	44	14	31,8
2022	43	48	26	54,2
2023	10	26	17	65,4

### 3.2 Rate of parasitization

**Figure 1:**
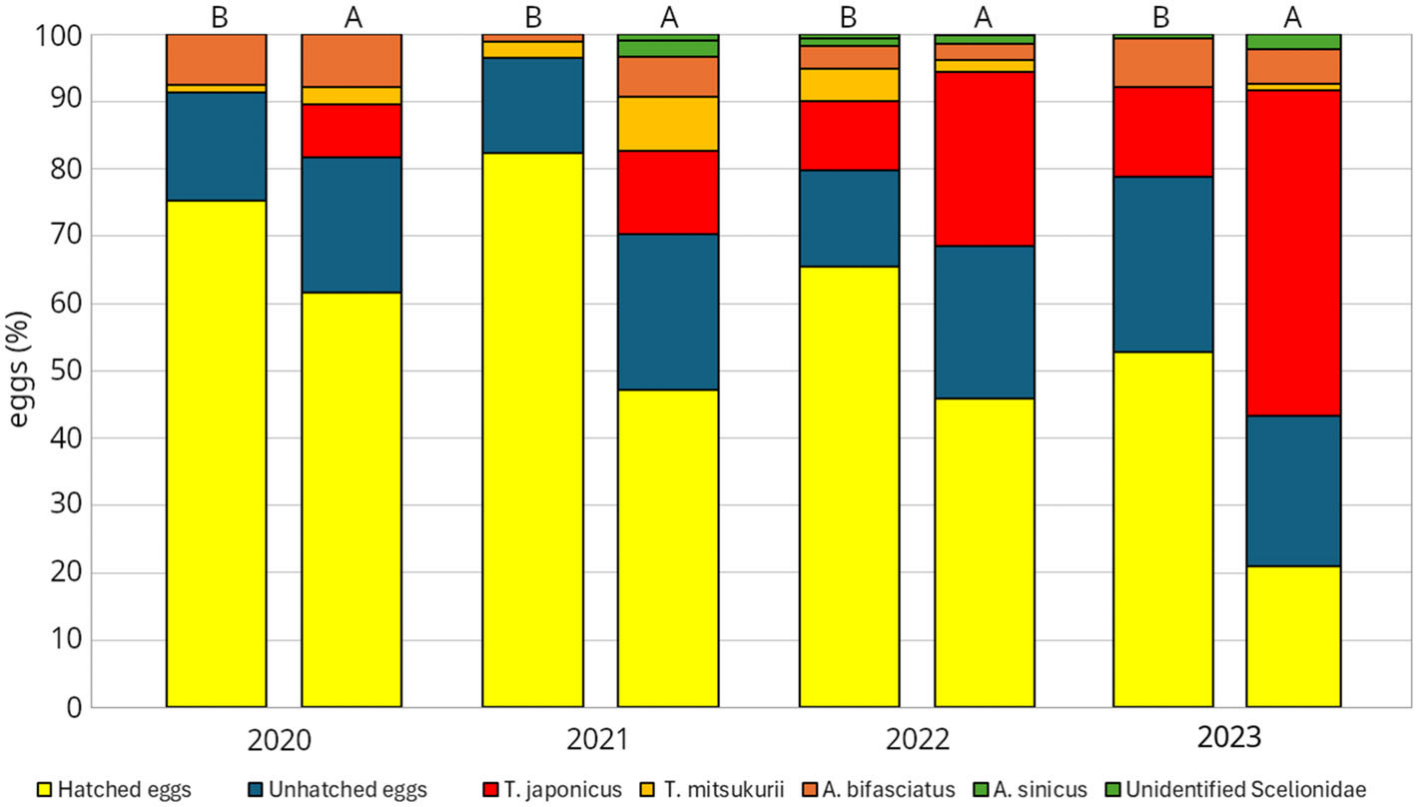
Percentage of BMSB eggs hatched, unhatched and parasitized by the three main parasitoid species *T. japonicus*, *An, bifasciatus* and *T. mitzukurii* and the impact of the hyperparasitoid *Acroclisoides sinicus* before (B) and after (A) the releases, for each year, from 2020 to 2023, in the establishment sites.

As shown in Fig. 1, *T. japonicus* had not been found at the release sites before the start of the program in 2020. The discovery of *T. japonicus* before the release occurred only 2 years after the program began, confirming its ability to survive the winter, as already demonstrated in the previous study.^19^ The overall rate of egg parasitization increased significantly over the 4 years of the program, from 18.3% to 50.6% (Z = 25.4, *P* < 0.001) (Fig. 1). The contribution of native natural enemies suffered a significant reduction: abundance of *An. bifasciatus* reduced from 7.8% in 2020 to 2.6% in 2022 followed by 6.1% increase in 2023 (Z = −7.1, *P* < 0.001); and abundance of *T. mitsukurii* decreased from 2.6% to 1.6% (Z = −8.0, *P* < 0.001) on the total number of available eggs. On the contrary, a significant increase (Z = 34.5, *P* < 0.001) in the parasitization rate by *T. japonicus* was observed, rising from 7.9% in 2020 to 41.2% in 2023. In particular, the contribution of *T. japonicus* within the total parasitization rate significantly increased (Z = 10.7, *P* < 0.001) in the same lapse time from 43% to 82%, a value already reached in 2022 and confirmed in 2023. The hyperparasitoid *Acroclisoides sinicus* (Hymenoptera: Pteromalidae) was also recovered from parasitized eggs (0.0–2.5%). In parallel, a significant reduction (Z = −24.3, *P* < 0.001) in the percentage of BMSB nymphs produced from collected eggs was also registered: it was 61.6% when the program started and dropped to as low as 29% after 4 years of the release program. A substantially constant percentage of eggs (range: 20.1–23.2%) remained unhatched (Z = 0.60, *P* = 0.27), in part due to predation and in part for unidentified causes.

### 3.3 Parasitization efficacy

The efficacy of parasitization by *T. japonicus* increased significantly during the release program, in terms of both discovery efficacy, which increased from 9.9% in 2020 to 54.4% in 2023 (Cochran‐Armitage test, Z = 8.3, *P* < 0.001), and parasitoid impact, which increased from 7.9% to 39.6% over the same period (Z = 32.1, *P* < 0.001) (Table 3).

**Table 3:** Indices of parasitization efficacy of *T. japonicus* (Tj)

	Egg masses (n)	Tj parasitizated egg masses (n)	Discovery efficacy	Eggs (n)	Tj parasitizated eggs (n)	Parasitoid impact
2020	121	12	9.9	3201	253	7.9
2021	122	23	18.9	3055	337	11.0
2022	289	115	39.8	7198	1790	24.9
2023	114	62	54.4	2555	1011	39.,6

### 3.4 Fruit damage

**Figure 2:**
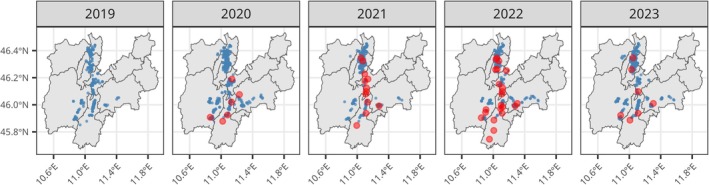
Distribution of the orchards monitored for fruit damage (blue dots) and the successful release sites where *T. japonicus* was recaptured in the same year (red dots) are reported.

Fruit injury at harvest was assessed as part of the field surveillance activities by sampling hanging apples in several ‘high‐risk’ orchards, which means they bordered woods, hedges, shrubs, and other crops that are potential sources of infestation. The scouting activity was performed by the advisory service personnel and the spatial distribution of the sampling points over the full Trento province is shown in Fig. 2. On the same plot we also highlighted the successful release sites.

The plot clearly highlights the spatial and temporal variability of the scouting activity even if, taking all years together, the release areas were evenly distributed across the different sites (Fig. S2).

**Figure 3:**
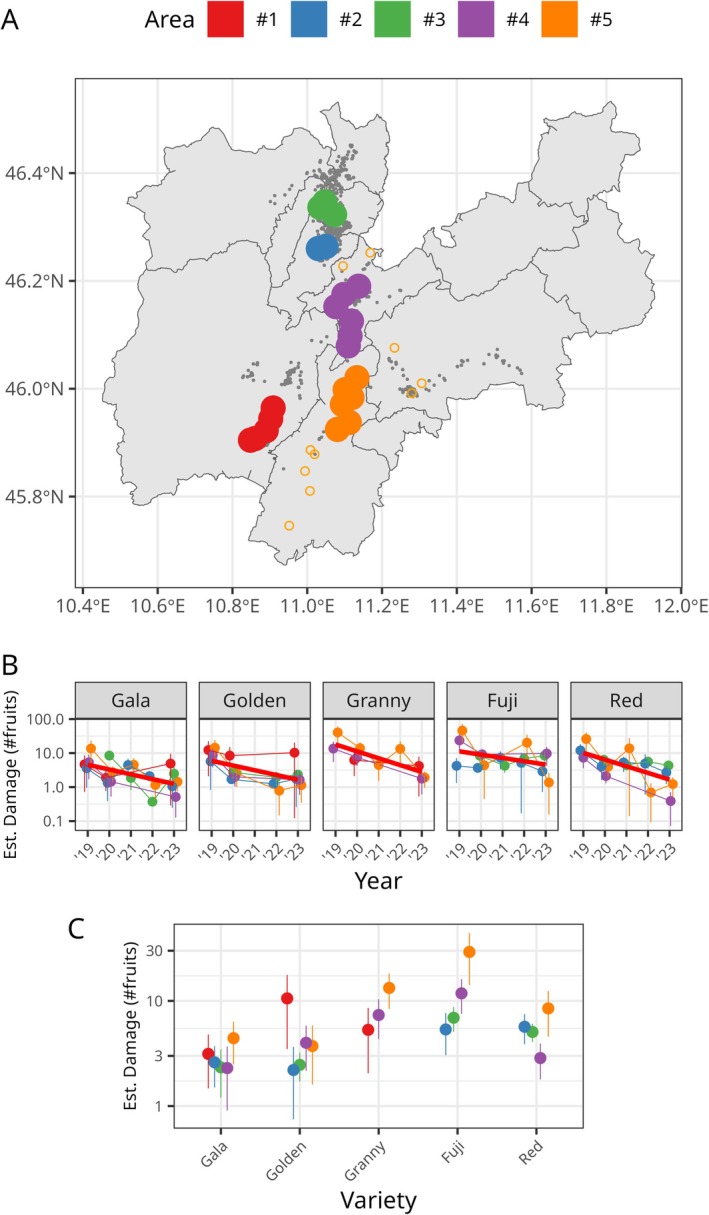
(A) Map showing the five focus areas. (B) Predicted fruit damage for the five main varieties across the 5 years. The vertical bars identify the 95% confidence intervals for the expected values. The red line highlights the general trends. (C) Estimated marginal effect for the variety. The vertical bars correspond to the 95% confidence intervals.

It is important to highlight that due to the characteristics of data collection, fruit damage assessment was observational. Sampling sites were not constant across the years, orchard management was not standardized, and it was impossible to assess/control the presence of the parasitoid. For these reasons, identifying a reliable set of control plots was not feasible. Instead, we opted to follow the trends in fruit damage ‘longitudinally.’ To analyze these trends, we identified five macro areas close to the release points (see MM), which showed a consistent number of monitoring sites over the years. Their location is shown in Fig. 3(A) and their overall characteristics are summarized in Table 1. Fig. 3(B) instead shows the results of the gam modeling in terms of the expected values for the damage over the 5 years for the different apple varieties in the five study areas. Each point in the figure represents the estimated expected damage for each variety in the five macro areas. The evolution of the damage on the individual areas is highlighted by colored lines, while a trend line is superimposed on the plots to highlight the global progression. Despite the expected variability in damage levels, a consistent decreasing trend is observed in all cases. It is important to highlight that the figure presents data on a logarithmic scale, meaning that a reduction of one unit corresponds to a tenfold decrease in damage. Even if due to the type of monitoring the trends cannot be unambiguously attributed to the parasitoid release alone, the presence of common and coherent decrease in the damage over time is clearly visible and the presence of a variety specific trend cannot be ruled out. To further highlight potential differences among the varieties, adjusted predictions for the expected damage were calculated for the five macro‐areas and are shown in Fig. 3(C). Here the dots represent the ‘average’ expected level of damage. The overall lower level of damage for Gala and Golden is clear and was observed almost everywhere with the sole exception of Area #1 for Golden. In general, however, estimation of the damage in Area #1 turned out to be less reliable due to the lower number of observations present in that area. In terms of variety susceptibility, our results based on large‐scale surveys confirmed the observation of previous studies carried out at the orchard level. Gala, being the earliest variety, showed the lowest level of injury. Among the mid‐season varieties, Granny Smith is confirmed to be more susceptible than Golden and not markedly different to the late‐season Fuji (Table S4).

## 4 DISCUSSION

The present study supports early observations^19^ of an increase in parasitism rates, providing robust data to support this trend. The overall egg parasitization rate reached 59.6% in 2023, surpassing the 36% reported for the native area.^38^ Additionally, the parasitism rate of *T. japonicus* increased from 7.9% in 2020 to 48.4% in 2023, with its contribution to the overall egg parasitization rate rising from 43% to 85% in the same period. Discovery efficacy and parasitoid impact peaked at 54.4% and 39%, respectively, values not previously observed in surveys determining the parasitization rate of BMSB eggs by native species either alone or combined with adventive or released alien species. A 3‐year survey in northern Italy reported a parasitization rate ranging from 12% to 21%, primarily due to the native species *Anastatus bifasciatus*.^56^ Early experiences of augmentative biocontrol releases of *An. bifasciatus* showed an average parasitism rate of sentinel BMSB egg masses ranging from 2% to 16%,^57^ while a more recent study reported a parasitization rate of 16.7% after a large‐scale augmentative biological control.^58^ While these studies agree that releasing *An. bifasciatus* increases the parasitization rate compared to control sites, they consider the control insufficient to efficiently suppress the pest.

The results of the present study also show that *T. japonicus* is extremely competitive, both in relation to the native species *An. bifasciatus* and the adventive species *T. mitsukurii*. The combined contribution of these two latter species to egg parasitization decreased significantly during the 4‐year survey. Studies on the potential competition between BMSB egg parasitoids have been conducted to evaluate the consequences of either augmentative biocontrol with *An. bifasciatus* or classical biological control with *T. mitsukurii* or *T. japonicus*. Among native species, *An. bifasciatus* is reported to be a superior intrinsic competitor compared to *Ooencyrtus telenomicida* Vassiliev (Hymenoptera Encyrtidae), with no or minor negative consequences expected when released together.^59^ Competition studies are often conducted to elucidate the interactions between native and exotic parasitoids that do not have a common evolutionary history. For example, one study reported that *T. japonicus* is more aggressive and defends egg masses much longer than the native species *T. cultratus*, proving to be a superior extrinsic competitor.^60^ Adult and larval interspecific competition between *T. japonicus* and *An. bifasciatus* was also assessed in another study,^61^ suggesting that the two parasitoids engage in counterbalance competition, with the former being a superior extrinsic competitor (egg guarding and aggressiveness) and the latter being a superior intrinsic competitor (successful development from multiparasitized eggs of all ages). The authors concluded that the two species can coexist in the same site and potentially show synergism in controlling BMSB. In other cases, competition studies aim to evaluate the consequences of releasing the selected species for CBC on the adventive populations of alien parasitoids. Studies conducted under controlled conditions showed that *T. mitsukurii* displayed more aggressive behavior and spent more time defending the host egg mass than *T. japonicus* when released simultaneously.^62^, ^63^ These findings contrast with field observations,^19^ where the efficacy of *T. mitsukurii* was lower in sites where *T. japonicus* was successfully established. Differences between laboratory data and field observations could be due to the effect of temperatures. Life table parameters investigated in laboratory experiments under four constant temperatures between 16 °C and 31 °C suggest that the population of *T. japonicus* is expected to be favored over *T. mitsukurii* at higher temperatures.^64^

Contrary to expectations based on competition studies conducted in laboratory experiments, *T. japonicus* is outcompeting both potential rivals in the open environment and expanding its presence, as indicated by the increasing number of sites where it was recaptured. The continuous increase in the parasitization rate of *T. japonicus* observed over the 4 years has largely compensated for the reduced contribution of *An. bifasciatus* and *T. mitsukurii*.

Regarding fruit damage, our preliminary data suggest a trend of reduced fruit injury coinciding with the increasing prevalence of *T. japonicus* and a reduction in BMSB chemical control. However, if this reduction can be directly attributed to the action of the parasitoid alone cannot be deduced from our dataset. As far as the severity of fruit damage is concerned, it is reported that it depends on pest pressure and is influenced by both the cultivar and management practices^3^ and the risk of injury is related to the relative maturity of the fruit at the time of exposure.^65^ In terms of fruit injury trend over the period in the five study areas results of our study show a constant significant decrease for all the varieties. To the best of our knowledge, this is among the first large‐scale studies to observe a potential association between the establishment of the parasitoid and fruit damage trends. This result is supported by the fact that, during the same period, there was an expansion of untreated areas from 24.7% in 2020 to 61.6% in 2023, along with consistent specific treatment pressure (1.48–1.49 insecticides/ha, range 1–6) on the treated areas over the last 3 years (Table S2). Additionally, the average number of specific treatments applied over the more than 8000 ha monitored decreased from 1.32 insecticides/ha in 2020 to 0.57 insecticides/ha in 2023. While it is true that the largest untreated surface was observed in 2019, it is equally true that this resulted in the highest percentage of damage to the fruits. Fruit growers were unprepared to face the expansion of the infestation area in 2019 and only reacted in 2020 by increasing the treated area. At the regional level, the percentage of treated area has decreased, indicating that the area affected by the harmful presence of the pest has contracted. On the other hand, the pressure from treatments remains constant where the pest is still present, coinciding with the areas most affected by migration from marginal and wild areas more favorable to overwintering and proliferation. We are aware of the limitations of these data: (1) it is essentially self‐reported by the producers, (2) it does not account for the potential side effects of other insecticide treatments used for the control of other pests, (3) it represents an average figure for a fruit‐growing area that extends well beyond the specific areas where the parasitoid has established, (4) the high mobility of *T. japonicus* ^66^ precluded the establishment and maintenance of a geographically distinct and truly representative control area. Given the insect's dispersal capabilities, maintaining a separation between release and control sites over the 4‐year study period would have been impractical and likely compromised the integrity of the comparison. Nevertheless, the large number of verified annotations (just under 4000) partially compensates for these limitations and supports the specific data collected regarding the establishment of the parasitoid and the reduction of fruit damage.

## 5 CONCLUSIONS

Classical biological control has been evaluated as a promising method for long‐term management of alien invasive pests.^67^ In Europe, there is a long list of apple pests that have been targeted for control through classical biological control with varying degrees of success. Noteworthy examples include the control programs launched in the early decades of the twentieth century of the alien species *Eriosoma lanigerum* (Hausmann) using its parasitoid *Aphelinus mali* Haldeman, and the control of the San Jose scale *Quadraspidiotus perniciosus* (Comstock) using the parasitoid *Encarsia perniciosi* (Tower). Despite their permanent establishment, control is not complete, and chemical intervention is needed for both pests depending on seasonal weather conditions and varietal susceptibility. This is likely due to the fact that in both cases, the pests are primarily, if not exclusively, associated with apple trees, and their ecological balance with their antagonists is strongly influenced by the management practices of the crop. The present program targets a polyphagous pest, whose large populations develop in the study areas mainly on wild hosts and migrate to the crops once the wild food becomes scarce. Results of the present large‐scale study show that *T. japonicus* was recaptured in the majority of the release sites and significantly contributed to the increase in the egg‐parasitization rate of BMSB in the sites of release reducing the pest pressure on the nearby apple orchards. Due to the nature of the damage dataset, however, the significant decrease in fruit injury cannot be attributed solely and unambiguously to biological control, but it clearly highlights a general trend which will require further confirmation. Other factors, such as climate conditions during the summer season may have also played a role in this positive trend; during the wettest seasons, such as in 2021, BMSB tend to stay longer in woodlands, taking advantage of the abundance of wild fruit instead of moving into crops in search of juicy fruits. Additionally, the improvement in growers' management competencies, particularly in assessing the risk of injury and in timing insecticide treatments, may have contributed to more effective chemical control of the pest damage.

Even though the results cannot be claimed as conclusive, the extended duration of the study and the large number of observations support attributing a strong role to biological control and declaring this experience a success. The positive outcome of this initiative was undoubtedly aided by the careful selection of release sites, predominantly consisting of natural areas minimally contaminated by insecticide residues. The objective was to establish the parasitoid in wooded areas at the edges of orchards to control efficiently and permanently BMSB populations before they migrated into the crops.

The results obtained also allowed us to supplement laboratory observations on the behavior of *T. japonicus*, particularly in relation to its greater competitiveness against the native species *An. bifasciatus* and the adventive populations of *T. mitsukurii*. This is a consequence of the more restricted host range of *T. japonicus* compared to the generalist parasitoid *An. bifasciatus* ^68^ and the oligophagous *T. mitsukurii*.^36^, ^69^ This limited host range, combined with its intrinsic and extrinsic competitive behavior, confirms *T. japonicus* as the best choice for implementing a classical biological control program to manage BMSB in apple orchards. Further research with a specifically designed sampling protocol is required to establish a causal link between *T. japonicus* and reduced fruit damage and to fully evaluate the overall impact of this biocontrol program.

## CONFLICT OF INTEREST

The authors declare no conflicts of interest.

## AUTHOR CONTRIBUTIONS

Claudio Ioriatti and Gianfranco Anfora conceived research and provided the funding, Livia Zapponi, Serena Chiesa and Gino Angeli coordinated the field scouting and insect identification. Pietro Franceschi and Valerio Mazzoni analyzed data. Claudio Ioriatti and Valerio Mazzoni drafted the manuscript. All authors reviewed, edited and approved the manuscript.

## Supporting information


**Data S1:** Supporting Information.

## Data Availability

The data that support the findings of this study are available from the corresponding author upon reasonable request.
