# Supplementary material for: Classical biological control of the brown marmorated stink bug (Halyomorpha halys) in apple orchard: a success story
Source: Pest Manag Sci. 2025 Aug 25;81(12):8500–9. doi: 10.1002/ps.70154 (PMC12618914; doi:10.1002/ps.70154)

**Supplementary materials**

Table 1S. Insecticide materials registered for the BMSB control during the period 2019-2023.

| A.I. | Trade name | Rate | Max applications/year | registration |
| --- | --- | --- | --- | --- |
| Acetamiprid | Epik SL, | 0.5-2 | 2 | registered |
| Acetamiprid | Kestrel, | 1,5 L/Ha | 2 | registered |
| Acetamiprid | Gazelle | 2 kg/ha | 2 | registered |
| Etofenprox | Trebon UP | 0.75 L/ha | 3 | registered |
| Deltametrina | Decis EVO | 0.7 L/ha | 1 | registered |
| Piretrine | Pyganic 1.4 | 2.5 L/ha | 2 | registered |
| Tau-fluvalinate | Mavrik smart | 0.6 l/ha | 2 | registered |
| Triflumuron | Alsystin | 0.375 L/ha | 2 | registered until 2021 |
| Fosmet | Spada 50 WG | 1.5 kg/ha | 1 | registered until 2021 |

Tab.2S. Number of treatments and percentage of treated area during the period 2019-2023 as resulted from the official field books of the apple grower members of the cooperative system (APOT).

|  | Field books | Monitored Surface | Treated surface | | average treatments/total monitored surface | average treatment/treated surface |
| --- | --- | --- | --- | --- | --- | --- |
| year | (n. of farms) | (ha) | (ha) | % | (n.) | (n.) |
| 2019 | 3940 | 8157.2 | 2433.2 | 29.8 | 0.38 | 1.28 |
| 2020 | 3882 | 8196.2 | 6173.2 | 75.3 | 1.32 | 1.75 |
| 2021 | 3805 | 8124.2 | 3969.8 | 48.9 | 0.72 | 1.48 |
| 2022 | 3764 | 8145.8 | 3568.8 | 43.8 | 0.64 | 1.48 |
| 2023 | 3715 | 8122.8 | 3118.9 | 38.4 | 0.57 | 1.49 |

## Table 3S. Number of fruit samples included in the statistical analysis for determining the impact on the fruit injury of the BMSB infestation in the five selected sub-areas.

## Trento North

| year | Fuji | Gala | Golden | Granny | Red |
| --- | --- | --- | --- | --- | --- |
| 2019 | 6 | 6 | 10 | 4 | 8 |
| 2020 | 21 | 9 | 16 | 20 | 38 |
| 2023 | 8 | 6 | 6 | 5 | 3 |

## Trento South

| year | Fuji | Gala | Golden | Granny | Red |
| --- | --- | --- | --- | --- | --- |
| 2019 | 9 | 12 | 4 | 9 | 7 |
| 2020 | 2 | 15 | 7 | 12 | 11 |
| 2021 | 0 | 16 | 0 | 8 | 3 |
| 2022 | 5 | 10 | 3 | 4 | 3 |
| 2023 | 2 | 18 | 10 | 16 | 9 |

## Arco

| year | Gala | Golden | Granny |
| --- | --- | --- | --- |
| 2019 | 11 | 6 | 0 |
| 2020 | 19 | 4 | 12 |
| 2023 | 4 | 6 | 10 |

## Low Non Valley

| year | Fuji | Gala | Golden | Red |
| --- | --- | --- | --- | --- |
| 2019 | 4 | 3 | 3 | 13 |
| 2020 | 6 | 12 | 8 | 21 |
| 2021 | 15 | 14 | 0 | 18 |
| 2022 | 2 | 2 | 3 | 2 |
| 2023 | 6 | 8 | 8 | 13 |

## Middle Non Valley

| year | Fuji | Gala | Golden | Red |
| --- | --- | --- | --- | --- |
| 2020 | 14 | 1 | 34 | 40 |
| 2021 | 26 | 13 | 0 | 51 |
| 2022 | 8 | 1 | 9 | 22 |
| 2023 | 33 | 14 | 27 | 42 |

Table 4S. Fruit varieties pairwise comparisons in the five subareas: those highlighted in red are statistically significant (P<0.05).

## Trento North

| term | estimate | std.error | statistic | p.value | s.value | conf.low | conf.high |
| --- | --- | --- | --- | --- | --- | --- | --- |
| Gala - Red | -0.56 | 0.87 | -0.65 | 0.52 | 0.95 | -2.26 | 1.14 |
| Gala - Granny | -5.05 | 1.66 | -3.04 | 0.00 | 8.70 | -8.30 | -1.79 |
| Gala - Golden | -1.70 | 1.15 | -1.48 | 0.14 | 2.85 | -3.95 | 0.55 |
| Gala - Fuji | -9.50 | 2.26 | -4.21 | 0.00 | 15.23 | -13.93 | -5.07 |
| Red - Granny | -4.48 | 1.61 | -2.79 | 0.01 | 7.58 | -7.63 | -1.34 |
| Red - Golden | -1.14 | 1.05 | -1.08 | 0.28 | 1.85 | -3.19 | 0.92 |
| Red - Fuji | -8.94 | 2.22 | -4.02 | 0.00 | 14.08 | -13.29 | -4.58 |
| Granny - Golden | 3.35 | 1.75 | 1.91 | 0.06 | 4.16 | -0.09 | 6.78 |
| Granny - Fuji | -4.45 | 2.60 | -1.71 | 0.09 | 3.52 | -9.55 | 0.65 |
| Golden - Fuji | -7.80 | 2.31 | -3.38 | 0.00 | 10.43 | -12.33 | -3.28 |

## Trento South

| term | estimate | std.error | statistic | p.value | s.value | conf.low | conf.high |
| --- | --- | --- | --- | --- | --- | --- | --- |
| Golden - Gala | -0.70 | 1.39 | -0.50 | 0.62 | 0.70 | -3.43 | 2.03 |
| Golden - Red | -4.76 | 2.23 | -2.13 | 0.03 | 4.92 | -9.14 | -0.39 |
| Golden - Granny | -9.56 | 2.59 | -3.69 | 0.00 | 12.11 | -14.65 | -4.48 |
| Golden - Fuji | -25.49 | 7.74 | -3.29 | 0.00 | 9.99 | -40.65 | -10.33 |
| Gala - Red | -4.06 | 2.09 | -1.94 | 0.05 | 4.27 | -8.16 | 0.03 |
| Gala - Granny | -8.86 | 2.57 | -3.44 | 0.00 | 10.77 | -13.91 | -3.82 |
| Gala - Fuji | -24.79 | 7.71 | -3.21 | 0.00 | 9.57 | -39.91 | -9.67 |
| Red - Granny | -4.80 | 3.03 | -1.59 | 0.11 | 3.15 | -10.74 | 1.13 |
| Red - Fuji | -20.73 | 7.81 | -2.65 | 0.01 | 6.98 | -36.03 | -5.43 |
| Granny - Fuji | -15.92 | 7.87 | -2.02 | 0.04 | 4.54 | -31.35 | -0.50 |

## Arco

| term | estimate | std.error | statistic | p.value | s.value | conf.low | conf.high |
| --- | --- | --- | --- | --- | --- | --- | --- |
| Golden - Gala | 7.43 | 3.59 | 2.07 | 0.04 | 4.70 | 0.39 | 14.47 |
| Golden - Granny | 5.26 | 3.78 | 1.39 | 0.16 | 2.61 | -2.15 | 12.66 |
| Gala - Granny | -2.17 | 1.81 | -1.20 | 0.23 | 2.12 | -5.73 | 1.38 |

## Low Non Valley

| term | estimate | std.error | statistic | p.value | s.value | conf.low | conf.high |
| --- | --- | --- | --- | --- | --- | --- | --- |
| Red - Fuji | 0.32 | 1.48 | 0.22 | 0.83 | 0.27 | -2.57 | 3.22 |
| Red - Golden | 3.47 | 1.14 | 3.05 | 0.00 | 8.79 | 1.24 | 5.70 |
| Red - Gala | 3.07 | 1.07 | 2.87 | 0.00 | 7.92 | 0.97 | 5.17 |
| Fuji - Golden | 3.15 | 1.37 | 2.30 | 0.02 | 5.54 | 0.47 | 5.84 |
| Fuji - Gala | 2.75 | 1.27 | 2.16 | 0.03 | 5.02 | 0.25 | 5.24 |
| Golden - Gala | -0.40 | 0.93 | -0.44 | 0.66 | 0.59 | -2.22 | 1.41 |

## Middle Non Valley

| term | estimate | std.error | statistic | p.value | s.value | conf.low | conf.high |
| --- | --- | --- | --- | --- | --- | --- | --- |
| Red - Golden | 2.60 | 0.62 | 4.17 | 0.00 | 15.02 | 1.38 | 3.83 |
| Red - Fuji | -1.85 | 1.02 | -1.81 | 0.07 | 3.83 | -3.85 | 0.15 |
| Red - Gala | 2.73 | 0.75 | 3.64 | 0.00 | 11.85 | 1.26 | 4.20 |
| Golden - Fuji | -4.45 | 1.00 | -4.46 | 0.00 | 16.89 | -6.41 | -2.50 |
| Golden - Gala | 0.13 | 0.69 | 0.19 | 0.85 | 0.23 | -1.22 | 1.48 |
| Fuji - Gala | 4.58 | 1.08 | 4.25 | 0.00 | 15.50 | 2.47 | 6.69 |

Figure 1S. Details of release, monitoring and detection of T. japonicus in 53 sites in Trentino, Italy, during the four-year study. Grey-shaded cells indicate the action performed (R=release; M=monitoring; D=detection).

Figure 2S. Model diagnostic plots for the five subareas.

## Trento North


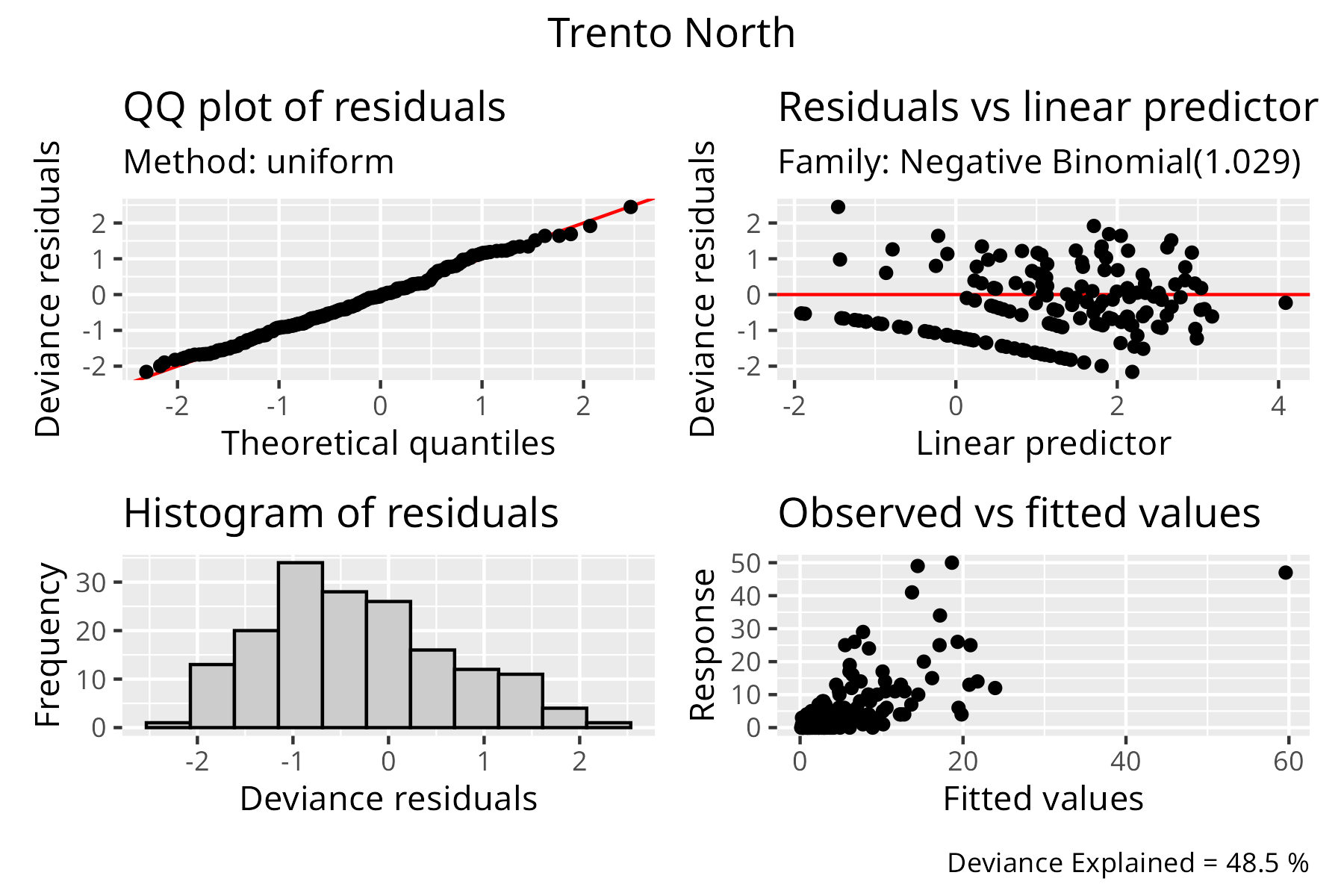


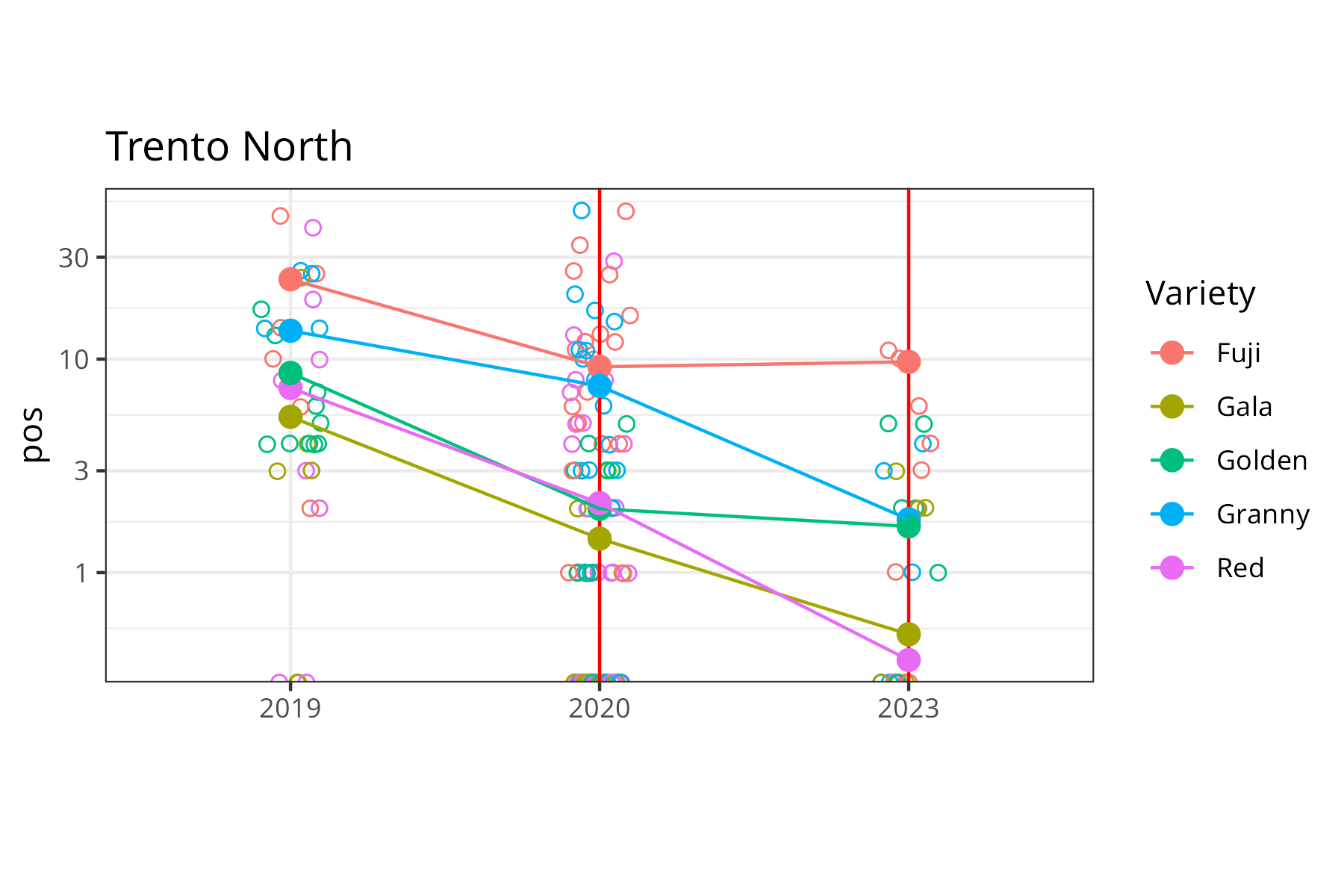


## Trento South


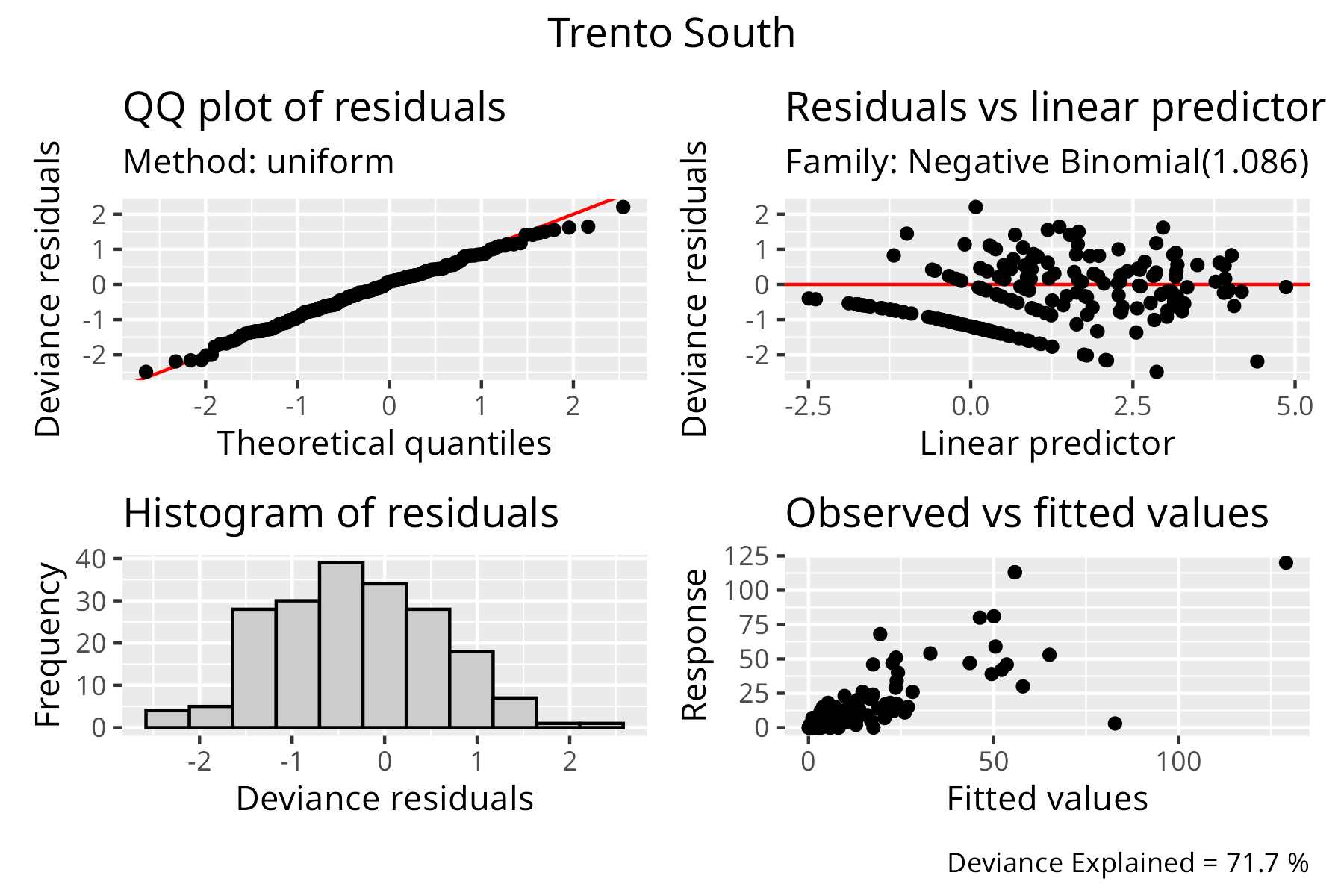


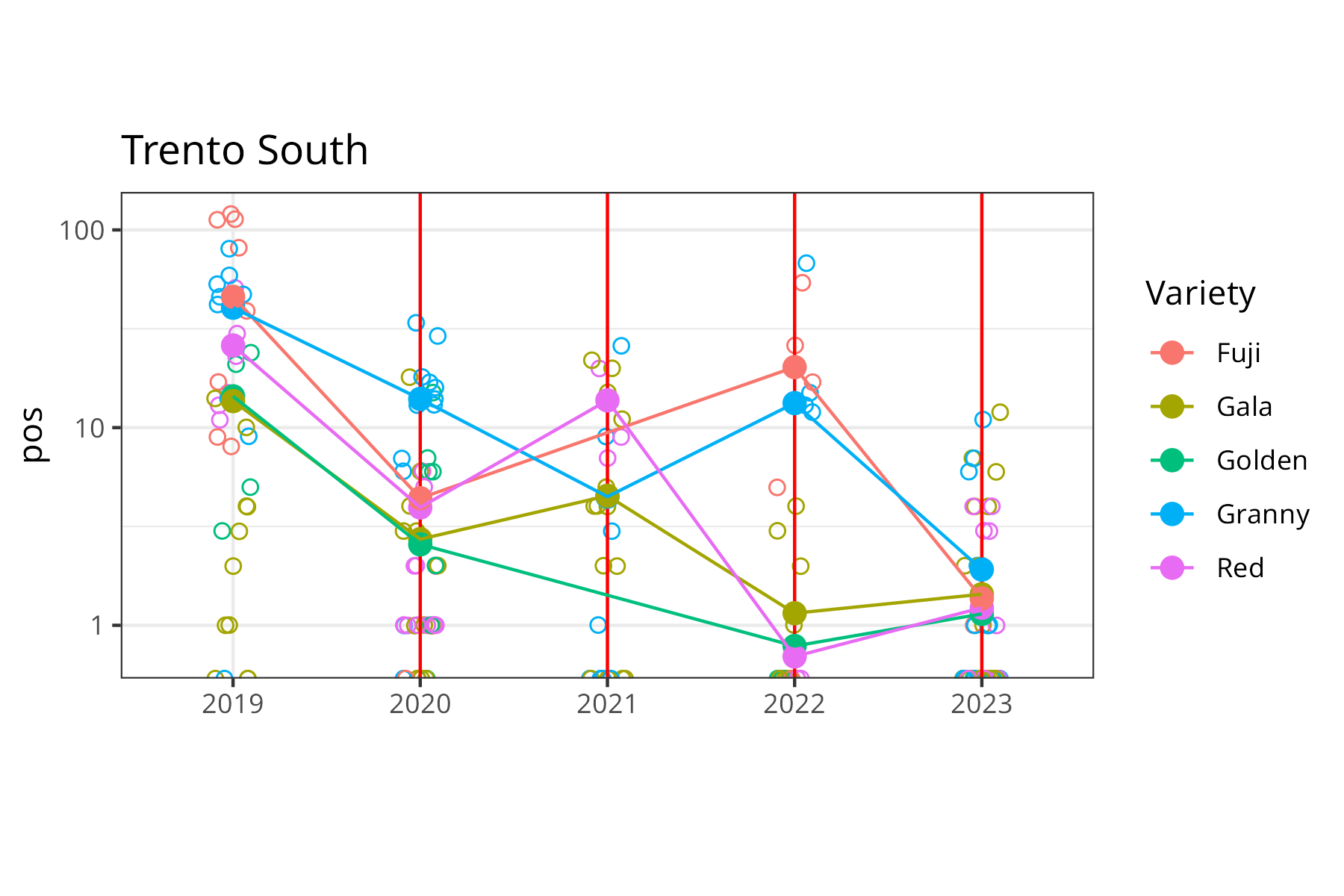


## Arco


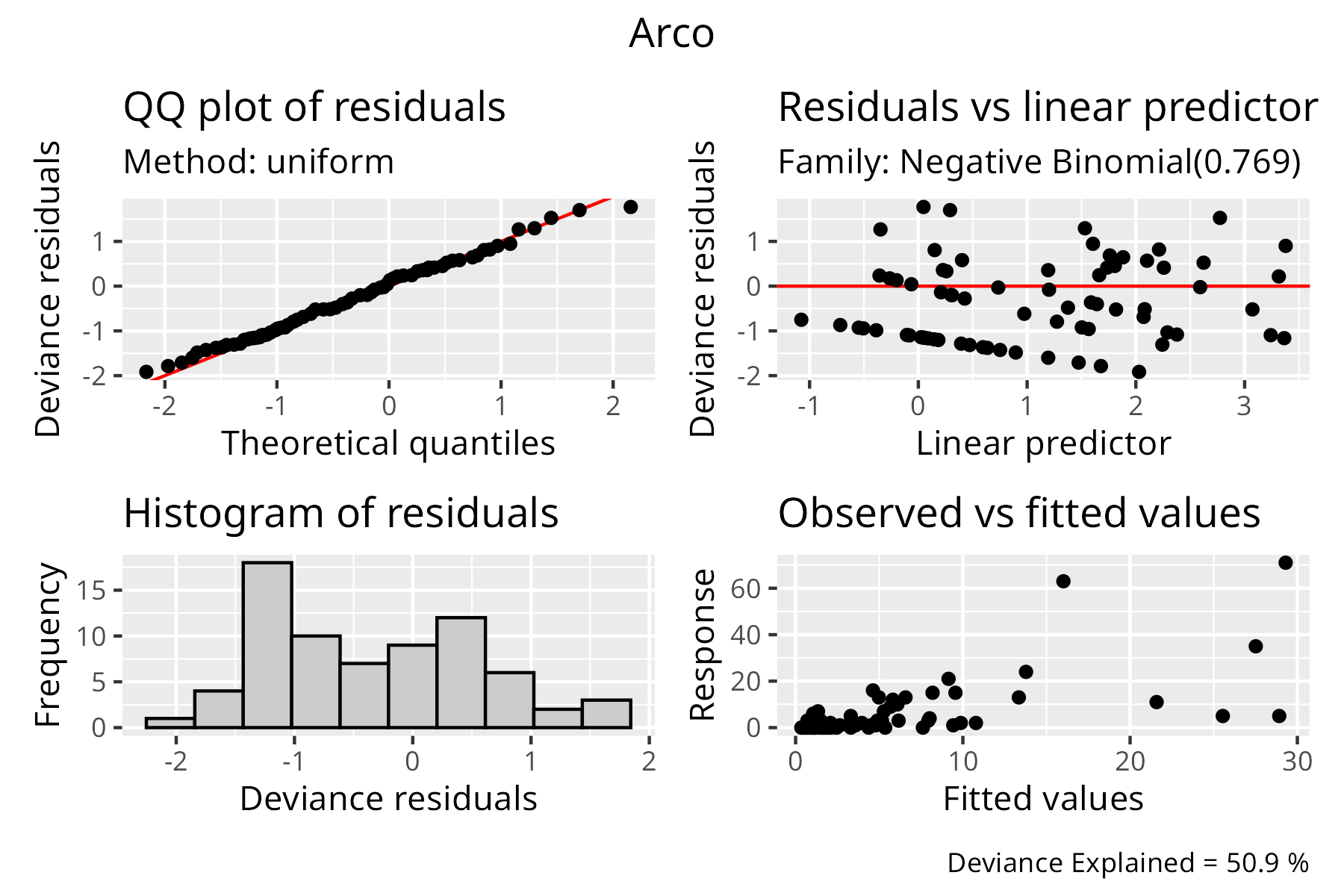


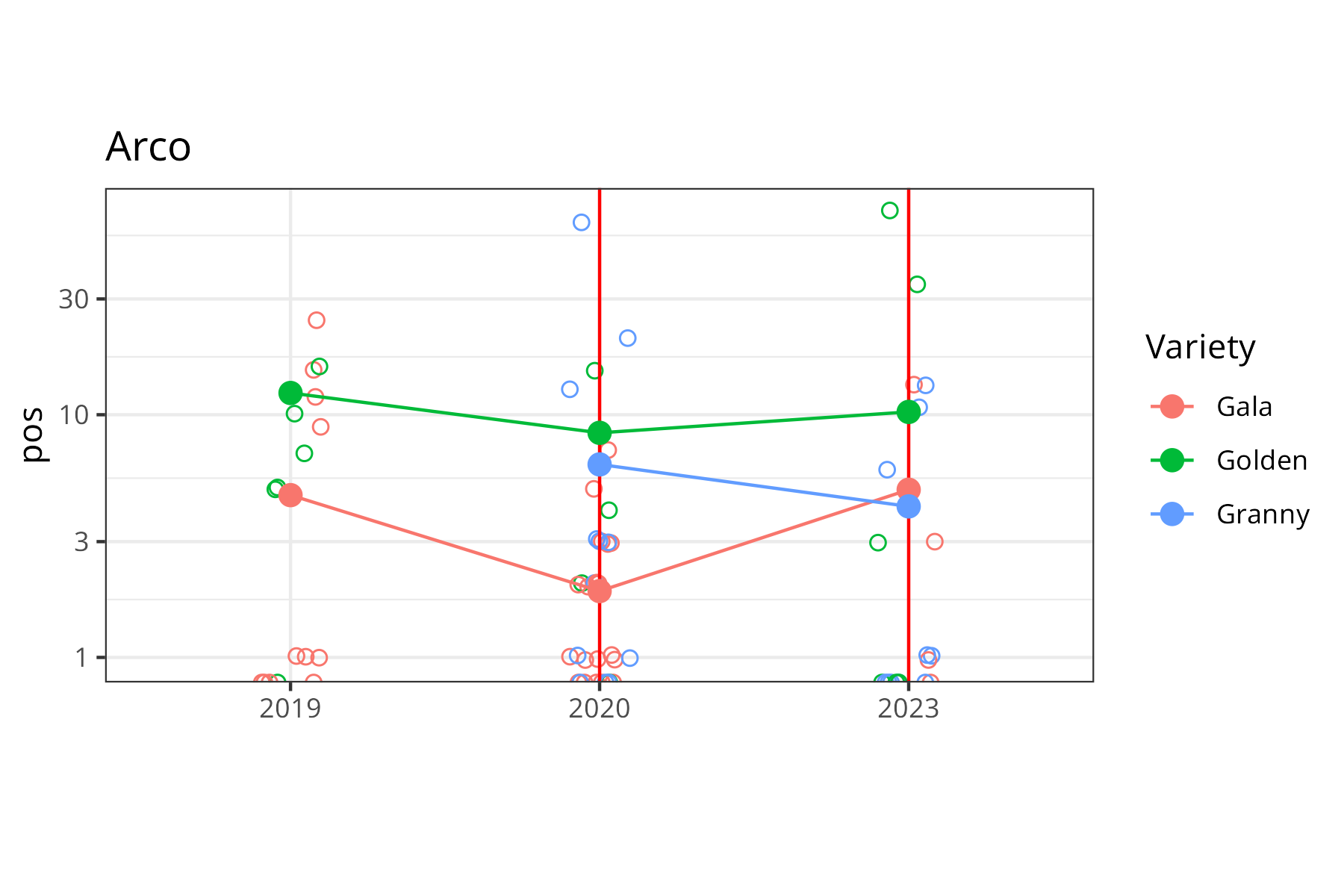


## Low Non Valley


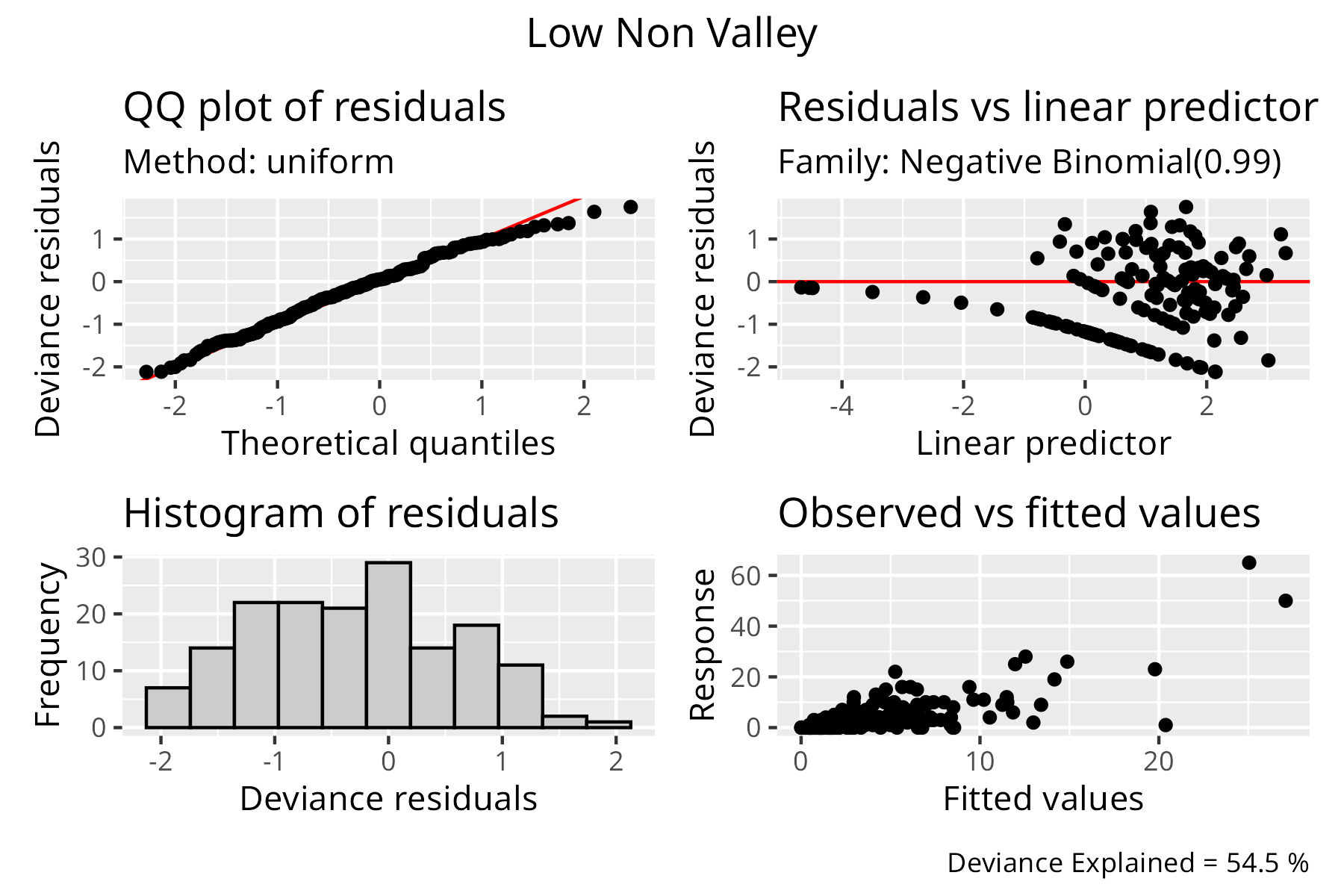


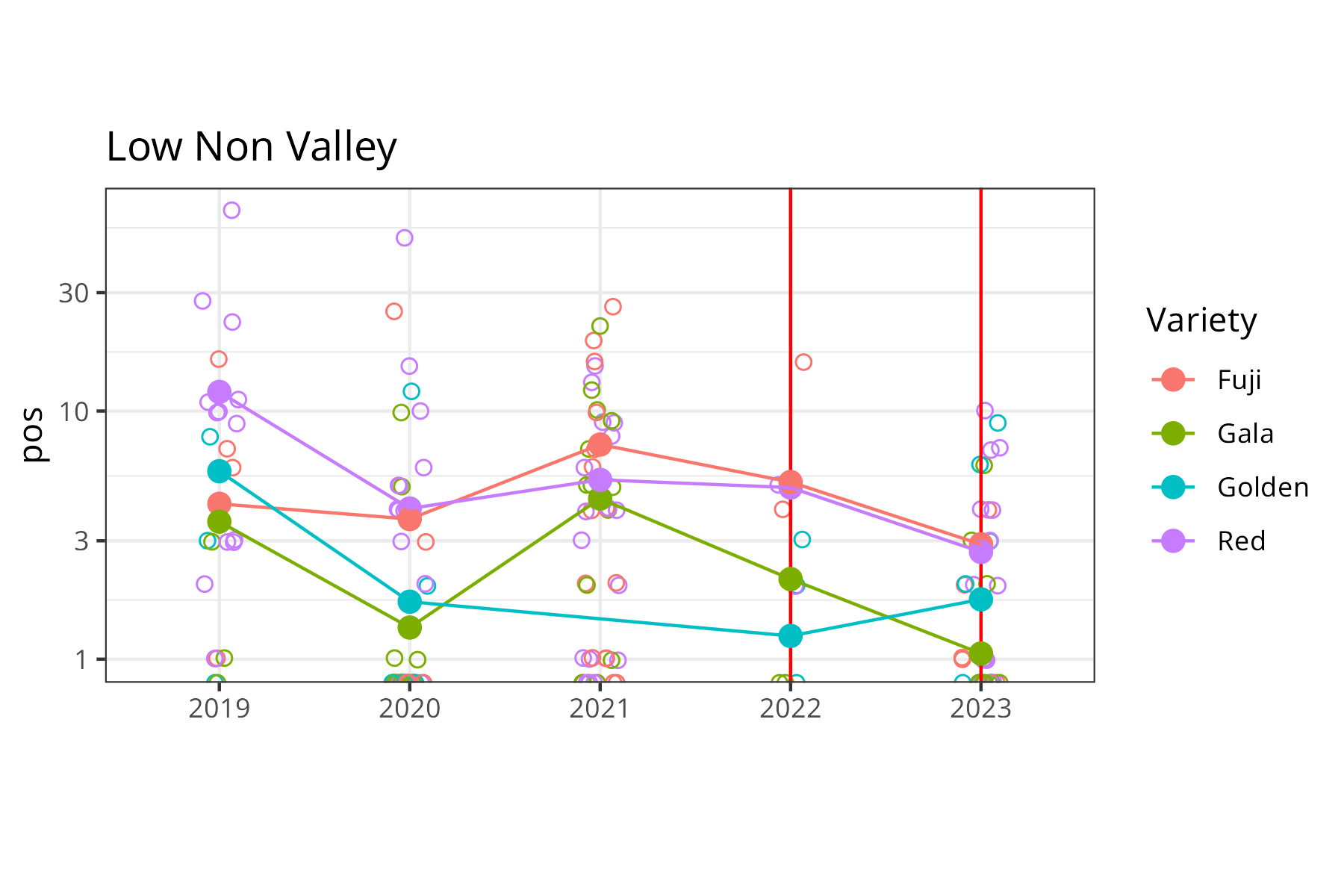


## Middle Non Valley


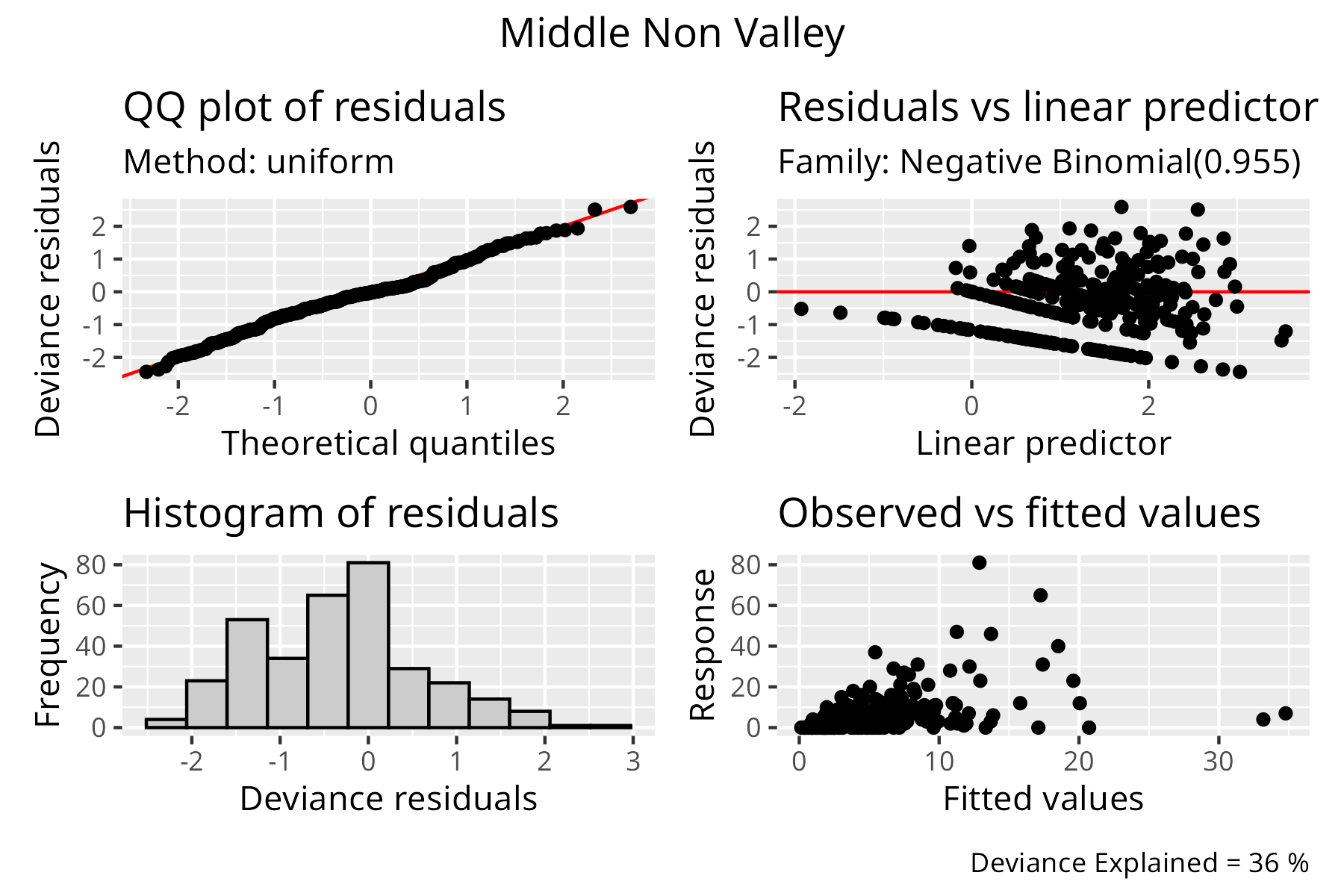


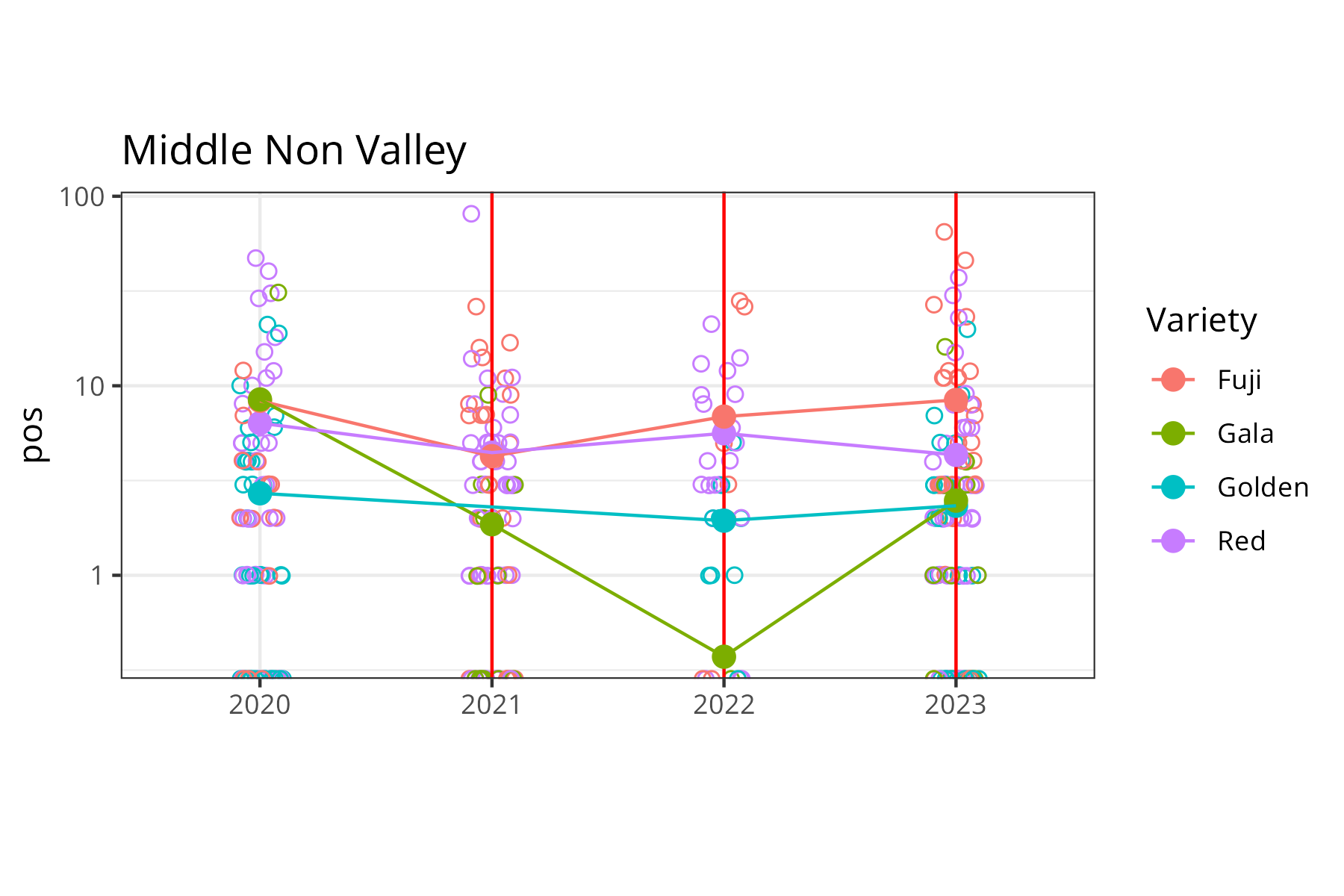

Supplement: Supplementary file 1 — Data S1: Supporting Information. [file PS-81-8500-s001.docx]
